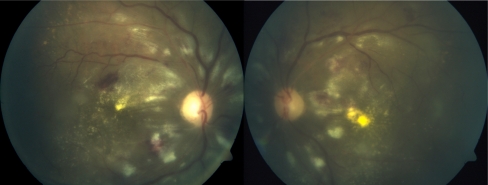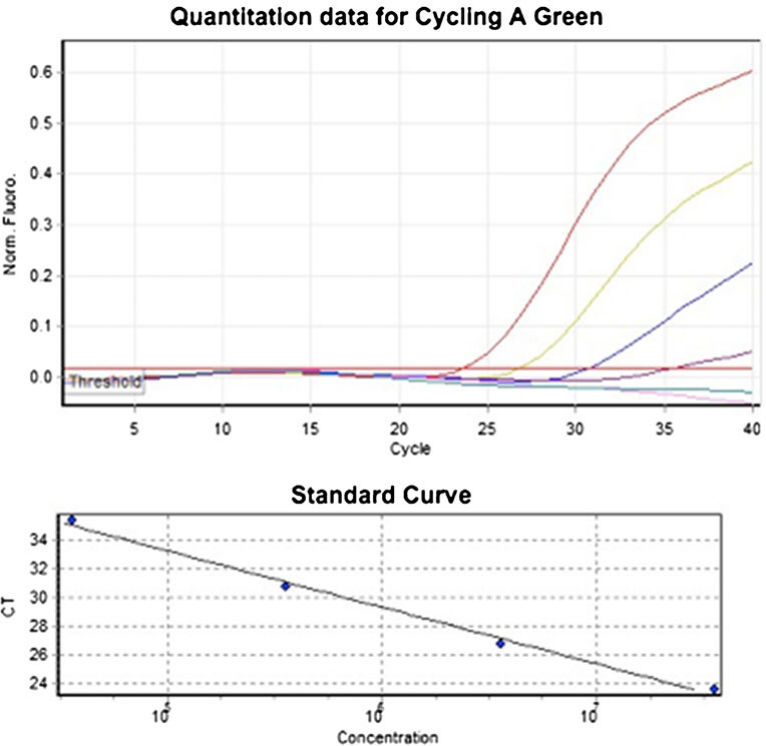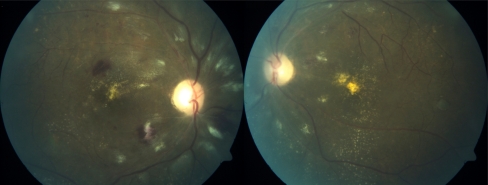# A case of bilateral Chikungunya neuroretinitis

**DOI:** 10.1007/s12348-011-0038-6

**Published:** 2011-09-01

**Authors:** Akshay Gopinathan Nair, Jyotirmay Biswas, Muna P. Bhende

**Affiliations:** Sankara Nethralaya, A Unit of Medical Research Foundation, 18 College Road, Nungambakkam, Chennai, 600 006 Tamil Nadu India

**Keywords:** Neuroretinitis, Retinitis, Chikungunya

A 65-year old Asian–Indian presented with a month-old history of sudden, painless diminution of vision in both eyes (OD—20/200, OS—20/400). A nondiabetic, he had no history of hypertension or tuberculosis. He gave a history of fever and malaise associated with joint pain 1 week prior to onset of ocular symptoms. Left eye showed 2+ cells in the anterior chamber, while the right eye was quiet. No afferent pupillary defect was noted. Dilated fundus examination revealed neuroretinitis, cotton wool spots, hemorrhages, and a grade 2 vitreous haze in both eyes (a). Investigations for antinuclear antibodies, C-reactive protein, HIV, hepatitis B and C, Widal, Mantoux and QuantiFERON TB Gold were negative. The total leukocyte count and ESR were raised. Serum angiotensin-converting enzyme levels as well as platelet count were within normal limits. Reverse transcription polymerase chain reaction assay for Chikungunya performed on the patient's serum revealed 358 copies of RNA/μl (b). A diagnosis of Chikungunya neuroretinitis was made, and the patient was treated with oral steroids and oral acyclovir 800 mg five times a day for 3 weeks, after which partial resolution was noted (c).